# Concept of terahertz waveguide plasmon amplifier based on a metal groove with active graphene

**DOI:** 10.1038/s41598-022-26268-x

**Published:** 2022-12-23

**Authors:** Mikhail Yu. Morozov, Vyacheslav V. Popov

**Affiliations:** Kotel’nikov Institute of Radio Engineering and Electronics (Saratov Branch), RAS, 38 Zelenaya Street, Saratov, Russia 410019

**Keywords:** Nanophotonics and plasmonics, Electronic properties and devices

## Abstract

We propose a concept of terahertz waveguide plasmon amplifier based on a metal groove with active graphene. It is shown that the power amplification factor of the longitudinal-section magnetic (LSM) waveguide plasmon (normalized to its wavelength) near the cut-off frequency of this mode can exceed the amplification factor of the transverse magnetic (TM) plasmon in a layered graphene structure by more than four orders of magnitude for the same frequency. This is caused by the increase of the LSM plasmon wavelength near the cut-off frequency, smaller energy velocity of the LSM mode, and greater energy release from graphene for the LSM plasmon due to stronger lateral confinement of the LSM waveguide plasmon as compared to the TM plasmon in a layered graphene structure. We show that the enhancement of the LSM plasmon amplification factor near the cut-off frequency is a stronger effect than that due to screening of graphene.

## Introduction

The use of terahertz (THz) plasmonic waveguides based on graphene makes it possible to increase the density of elements on a chip in THz integrated circuits, since the plasmon field confinement is an order of magnitude smaller than the THz electromagnetic wavelength at the same frequency^1^.

Graphene is an atom thick monolayer of graphite possessing unique properties such as high mobility, linear energy spectrum of free charge carriers and zero energy band-gap^2^. Creation of the inverse population of free charge carriers in graphene at THz frequencies^3^ as well as the development of tools for excitation of highly confined graphene plasmons^1^ resulted in possibility of compensation losses and amplification of THz electromagnetic field in active graphene structures giving rise to various concepts of THz graphene lasers^4,5^ and plasmonic amplifiers^6–8^. It is known that efficiency of the optical pumping of graphene is rather low because graphene absorbs less than 2.3% of the incident optical pump power^9^. A concept of the diffusion pumping of graphene allows for creating the free carrier population inversion in graphene with greater efficiency^5,8^. The diffusion pumping of graphene through a narrow-gap black-As substrate can prevent heating of graphene^10,11^. Creation of the population inversion in graphene via the electron and hole injection in graphene was studied in^12^. It was shown that electron–hole injection in graphene can substantially reduce the pumping threshold^13^. The possibility of plasmon amplification and necessary conditions for the plasmon lasing regime in graphene pumped by the electron–hole injection was discussed in^14^.

Propagation of THz plasmons in layered structures and metal waveguides with two-dimensional electron systems, particularly with graphene, has been actively studied theoretically and experimentally throughout the last decade. The effect of a graphene monolayer on the propagation of transverse electric modes in a rectangular metal waveguide was theoretically studied in^15^. The influence of embedded graphene layer on the guided modes of optical layered waveguides was investigated in^16^. Dispersion characteristics and damping of waveguide modes^17^, and the magnetoplasmon-polariton excitations^18^ in the layered structures with gated two-dimensional electron systems and graphene were studied theoretically.

Active guiding of THz surface plasmons in layered structure with inversely populated graphene with optical^6^ and diffusion^8^ pumping was theoretically discussed. Amplification of THz plasmons in a pair of parallel active graphene layers was theoretically studied in^19^. We will show below in this paper that the amplification of plasmons in a waveguide with active graphene could be more efficient than that in the layered graphene structures due to strong transverse confinement of the plasmon field in a waveguide.

Open plasmonic waveguides are promising for transferring the electromagnetic signals in THz plasmonic integrated circuits. Waveguide plasmons are at an advantage because the field of such plasmons is fully confined in the plane transverse to the direction of the plasmon propagation. Propagation of the channel plasmons and their field structure in rectangular and triangular metal grooves were theoretically discussed^20,21^ and experimentally realized^22^. The transition region between bound and leaky mode ranges in a metal groove waveguide was explored in^23^. Amplification of plasmons in waveguide structures with inversely populated graphene has not been studied so far.

**Figure 1:**
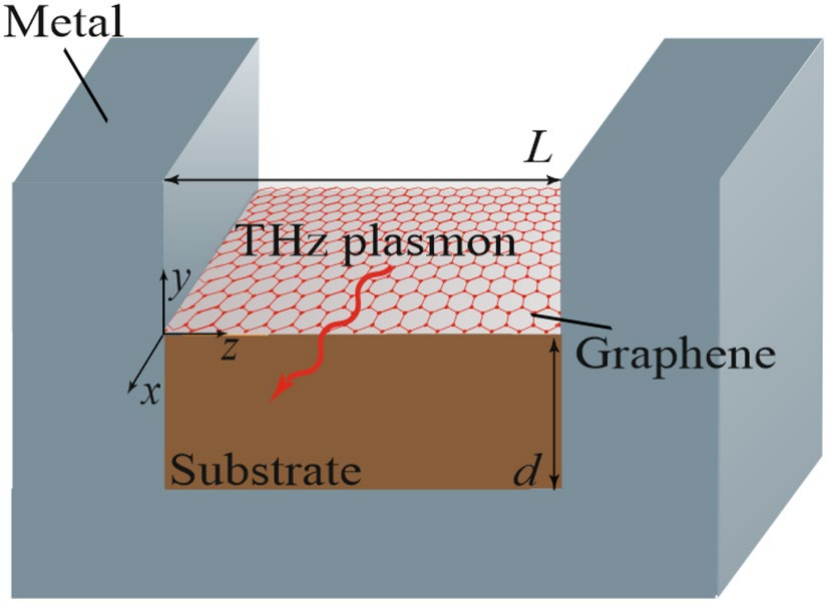
Schematic view of the structure under consideration.

In this paper, a concept of THz waveguide plasmon amplifier based on a metal groove with active (inverted) graphene, schematically shown in Fig. 1, is proposed. Inversely populated graphene is deposited on a dielectric substrate with the dielectric permittivity $\varepsilon_{{\text{s}}}$ and thickness *d*, placed in the bottom of a rectangular metal groove of width *L* and infinite depth. As a matter of fact, we assume that above graphene there is a hollow (containing a vacuum with the dielectric constant $\varepsilon_{{\text{a}}} = 1$) plane-parallel metal waveguide placed vertically.

## Results

**Figure 2:**
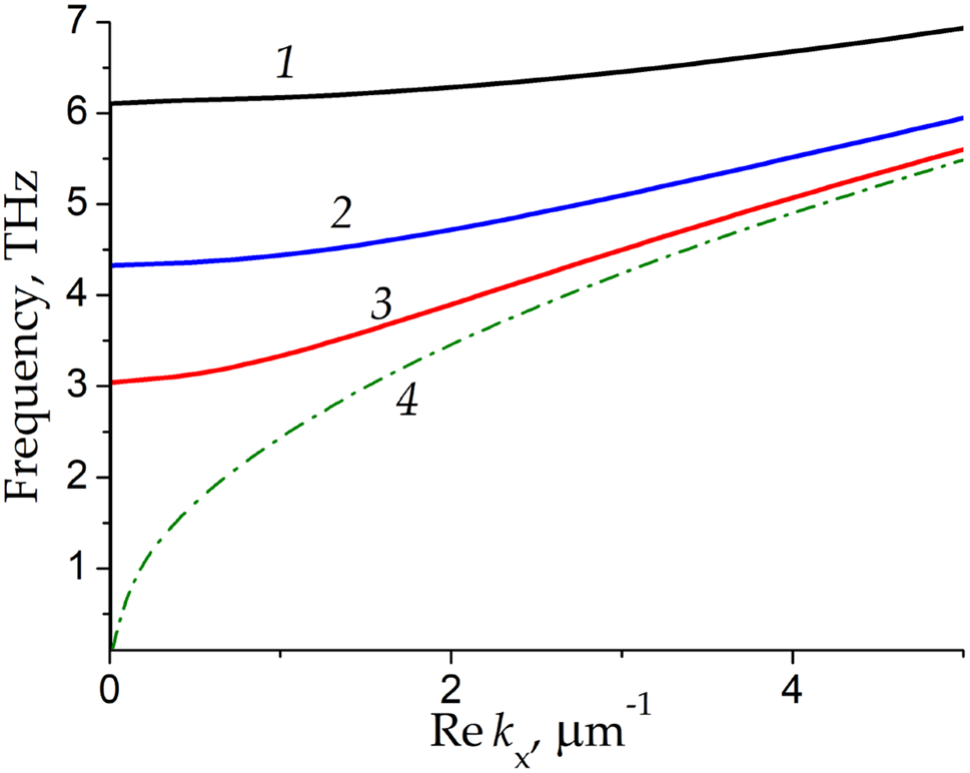
Dispersion of the LSM plasmon modes in the rectangular metal groove waveguide with active graphene for three different groove widths *L* = 0.5 μm, 1 μm, and 2 μm corresponding to curves *1*, *2*, and *3*, respectively, and TM plasmon mode dispersion in the infinite graphene sheet with a metal screen (dash-dotted curve *4*).

In a metal waveguide partially filled with dielectric, the eigenmodes of the waveguide split into the longitudinal-section magnetic (LSM) modes and the longitudinal-section electric (LSE) modes^24^ unlike the case of layered structures or hollow metal waveguides with transverse magnetic (TM) and transverse electric (TE) modes. We focus on the longitudinal-section magnetic (LSM) plasmon with the ac electric and magnetic fields $$\overset{\lower0.5em\hbox{$\smash{\scriptscriptstyle\rightharpoonup}$}}{{\text{E}}} \left( {E_{x} ,E_{y} ,E_{z} } \right)$$ and $$\overset{\lower0.5em\hbox{$\smash{\scriptscriptstyle\rightharpoonup}$}}{{\text{H}}} \left( {H_{x} ,0,H_{z} } \right),$$ respectively. Dispersion of the LSM plasmon in the rectangular metal groove waveguide with active graphene calculated by the dispersion relation presented in the Methods section is shown in Fig. 2 by curves *1*, *2*, and *3* corresponding to three different groove widths *L* = 1 μm, 2 μm, and 5 μm, respectively. Curve *4* in Fig. 2 corresponds to the TM plasmon mode in the infinite graphene sheet with a metal screen, having the ac fields $$\overset{\lower0.5em\hbox{$\smash{\scriptscriptstyle\rightharpoonup}$}}{{\text{E}}} \left( {E_{x} ,E_{y} ,0} \right)\;$$ and $$\overset{\lower0.5em\hbox{$\smash{\scriptscriptstyle\rightharpoonup}$}}{{\text{H}}} \left( {0,0,H_{z} } \right)$$^18^. Dispersion of the TM plasmon in a layered graphene structure and its increment were calculated using dispersion equation presented in^25^. One can see t hat, for large metal groove width, the LSM plasmon dispersion curve merges with the TM plasmon dispersion curve at high THz frequencies. This occurs because at high THz frequencies the influence of the side metal walls of the groove becomes negligible and the wavevector component transverse to the plasmon propagation direction becomes much smaller as compared to the longitudinal one: $$k_{x} > > k_{z} = {\pi \mathord{\left/ {\vphantom {\pi L}} \right. \kern-\nulldelimiterspace} L}.$$ With decreasing the groove width, the LSM and TM modes dispersion curves diverge. In distinction from the gapless TM plasmon, the LSM plasmon experiences the cut-off at finite frequency. The cut-off frequency of the LSM plasmon increases with decreasing the groove width.

**Figure 3:**
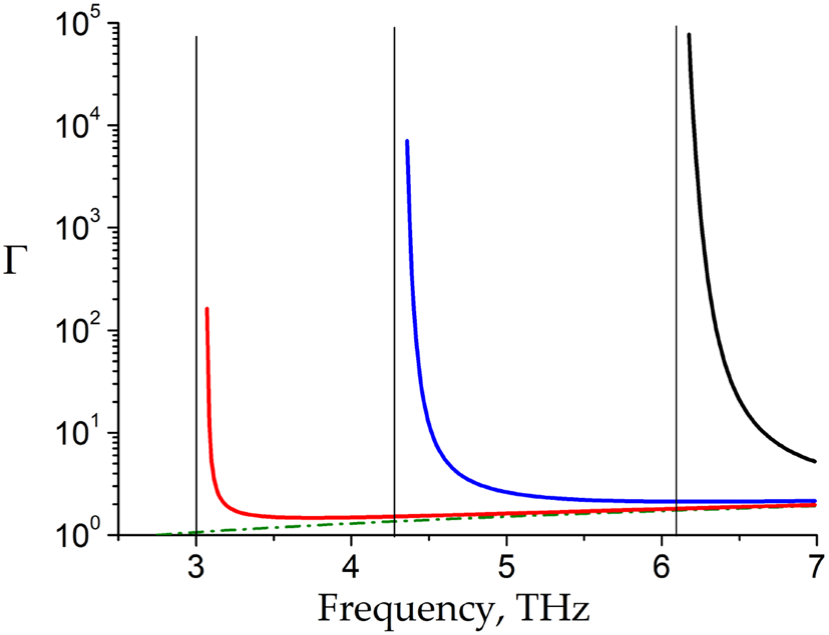
The power amplification factor Γ of the LSM plasmon in dependence on frequency for three different groove widths *L* = 0.5 μm, 1 μm, and 2 μm indicated by curves *1*, *2*, and *3*, respectively. The TM plasmon amplification factor is shown by dash-dotted curve *4*. Vertical straight thin lines indicate the cut-off frequencies of the LSM modes.

In Fig. 3, we demonstrate the power amplification factor of the LSM plasmon, normalized to the plasmon wavelength $\lambda = {{2\pi } \mathord{\left/ {\vphantom {{2\pi } {{\text{Re}} k_{x} }}} \right. \kern-\nulldelimiterspace} {{\text{Re}} k_{x} }}$ defined as $\Gamma = \exp \left( {\alpha \cdot \lambda } \right) = \exp \left( {{{ - 4\pi {\text{Im}} k_{x} } \mathord{\left/ {\vphantom {{ - 4\pi {\text{Im}} k_{x} } {{\text{Re}} k_{x} }}} \right. \kern-\nulldelimiterspace} {{\text{Re}} k_{x} }}} \right),$ which indicates the enhancement of the LSM plasmon power at the plasmon wavelength, for the same three groove widths *L* as in Fig. 2 (here $\alpha = - 2{\text{Im}} k_{x}$ is the plasmon power increment). At high THz frequencies the amplification factor of the LSM plasmon tends to the TM plasmon amplification factor likewise the dispersion curves of these modes behave in Fig. 2. At low THz frequencies, the increment of the LSM plasmon increases rapidly. One can see that near the cut-off frequency the power amplification factor of the LSM plasmon can exceed that of the TM plasmon by more than four orders of magnitude at the same frequencies. Note that we truncate the amplification factor curves (and all other curves below) at the points where the imaginary part of the longitudinal wavevector rises to become equal to its real part because the greater amplification factors are beyond the applicability of the linear theory used in our paper.

## Discussion

**Figure 4:**
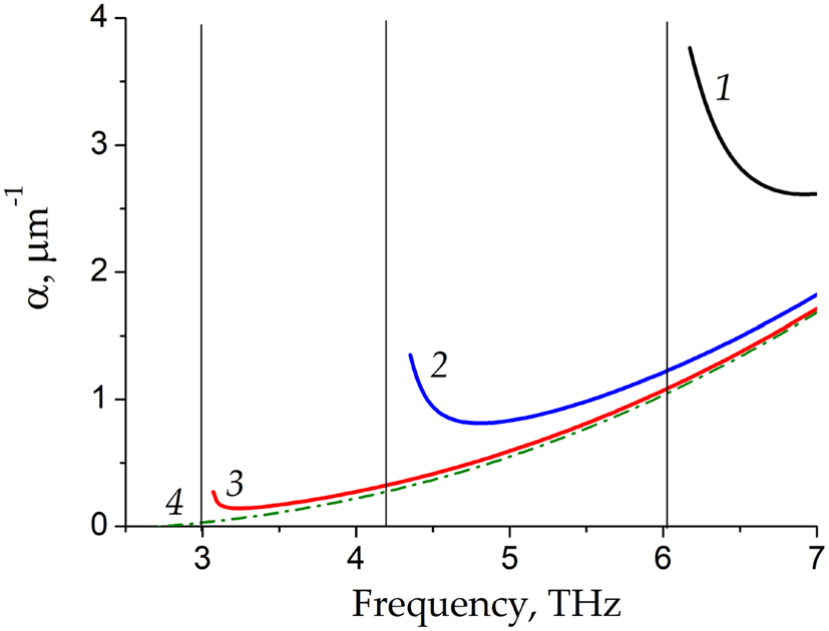
The power increment α in dependence on frequency for three different groove widths* L* = 0.5 μm, 1 μm, and 2 μm indicated by curves *1*, *2*, and *3*, respectively. The TM plasmon increment is shown by dash-dotted curve *4*. Vertical straight thin lines indicate the cut-off frequencies of the LSM modes.

Let us discuss the physical origins of significant increase of the LSM plasmon power amplification factor. First, the longitudinal component of the LSM plasmon wavevector $k_{x}$ tends to zero near the cut-off frequency leading to sharp increase of the LSM plasmon wavelength $\lambda = {{2\pi } \mathord{\left/ {\vphantom {{2\pi } {{\text{Re}} k_{x} }}} \right. \kern-\nulldelimiterspace} {{\text{Re}} k_{x} }}.$ Second, the power increment of the LSM plasmon $\alpha = - 2{\text{Im}} k_{x}$, presented in Fig. 4, for the same three groove widths *L* as in Figs. 2 and 3, also increases near the cut-off frequency. At low THz frequencies, the power increment of the LSM plasmon can exceed that of the TM plasmon by several times at the same frequency.

We wonder which factors influence the increment of the modes. According to the energy conservation law, the spatial growths of the plasmon energy flux defined as

$$S_{x} = \frac{1}{2}\int\limits_{0}^{L} {\,\int\limits_{ - d}^{\infty } {{\text{Re}} \left[ {E_{y} \cdot H_{z}^{*} } \right]\,dy} \,dz}$$

is related to the value of the power density released from active graphene (per unit length of the waveguide) $P = - \frac{1}{2}{\text{Re}} \sigma_{{{\text{gr}}}} \left( \omega \right)\int\limits_{0}^{L} {\,\left( {\,\left| {E_{x} \left( {y = 0} \right)} \right|^{2} + \left| {E_{z} \left( {y = 0} \right)} \right|^{2} } \right)\,dz}$ as $P = {{dS_{x} } \mathord{\left/ {\vphantom {{dS_{x} } {dx}}} \right. \kern-\nulldelimiterspace} {dx}}$^19^, where $\sigma_{{{\text{gr}}}} \left( \omega \right)$ is the graphene conductivity discussed in the Methods section. Taking into account the spatial dependence of the plasmon field, the last formula can be transformed to $- 2{\text{Im}} k_{x} = {P \mathord{\left/ {\vphantom {P {S_{x} }}} \right. \kern-\nulldelimiterspace} {S_{x} }}.$ On the other hand, the ratio of the plasmon energy flux $S_{x}$ and the plasmon energy density (per unit length of the waveguide),

$$W = \frac{1}{4}\int\limits_{0}^{L} {\left\{ {\int\limits_{ - d}^{0} {\left[ {\varepsilon_{0} \varepsilon_{{\text{s}}} \,\left( {\sum\limits_{m = x,y,z} {\left| {E_{m} } \right|^{2} } \,} \right)\, + \mu_{0} \,\left( {\sum\limits_{m = x,z} {\left| {H_{m} } \right|^{2} } \,} \right)\,} \right]dy} } \right.} + \left. {\int\limits_{0}^{\infty } {\left[ {\varepsilon_{0} \varepsilon_{{\text{a}}} \,\left( {\sum\limits_{m = x,y,z} {\left| {E_{m} } \right|^{2} } \,} \right)\, + \mu_{0} \,\left( {\sum\limits_{m = x,z} {\left| {H_{m} } \right|^{2} } \,} \right)\,} \right]dy} } \right\}dz,$$

defines the energy velocity of the plasmon mode^26^ $v_{{\text{E}}} = {{S_{x} } \mathord{\left/ {\vphantom {{S_{x} } W}} \right. \kern-\nulldelimiterspace} W}.$ Hence the increment of the plasmon mode can be written as

1 $$- 2{\text{Im}} k_{x} = {1 \mathord{\left/ {\vphantom {1 {v_{{\text{E}}} }}} \right. \kern-\nulldelimiterspace} {v_{{\text{E}}} }} \cdot {P \mathord{\left/ {\vphantom {P W}} \right. \kern-\nulldelimiterspace} W}.$$

As can be seen from Eq. (1), the power increment of the plasmon mode $\alpha = - 2{\text{Im}} k_{x}$ depends on the plasmon energy velocity $v_{{\text{E}}}$ as well as on the ratio between the power density released from active graphene and the plasmon energy density ${P \mathord{\left/ {\vphantom {P W}} \right. \kern-\nulldelimiterspace} W}.$

**Figure 5:**
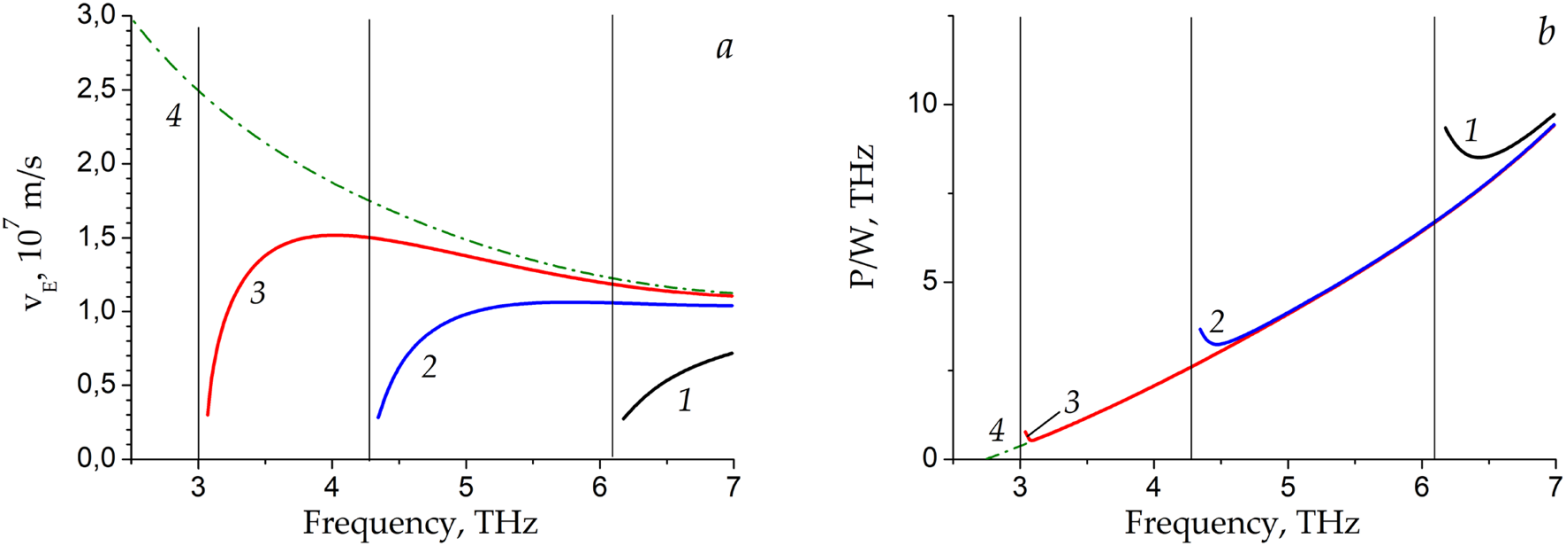
The plasmon energy velocity (**a**) and the ratio ${P \mathord{\left/ {\vphantom {P W}} \right. \kern-\nulldelimiterspace} W}$ between the power density released from active graphene and plasmon energy density (**b**) in dependence on frequency for three different groove widths *L* = 0.5 μm, 1 μm, and 2 μm indicated by curves *1*, *2*, and *3*, respectively. The energy velocity and the ratio ${P \mathord{\left/ {\vphantom {P W}} \right. \kern-\nulldelimiterspace} W}$ for the TM plasmon mode in the infinite graphene sheet with a metal screen are shown in panels *a* and *b* by dash-dotted curves *4*. Vertical straight thin lines indicate the cut-off frequencies of the LSM modes.

We calculated the energy velocities of the LSM plasmon presented in Fig. 5a for the same three groove widths *L* as in the previous figures in comparison with the energy velocity of the TM plasmon. One can see that the energy velocity of the TM plasmon monotonically increases with decreasing the plasmon frequency. The energy velocity of the LSM plasmon is smaller than the energy velocity of the TM plasmon and markedly decreases near the cut-off frequencies. Also note that the energy velocity of the LSM plasmon becomes smaller for smaller width of the groove. At the cut-off frequency, the plane wave oscillates strictly across the side walls of the groove waveguide being homogeneous (with zero longitudinal component of the wavevector) along the waveguide. As a result, the wave does not transfer the energy along the waveguide so that the energy velocity becomes zero in this case. In general, the cut-off frequency of a specific metallic waveguide mode is determined by the transverse wavevector of this mode^24^ which in turn depends on the width of the metal groove in our case.

The ratio ${P \mathord{\left/ {\vphantom {P W}} \right. \kern-\nulldelimiterspace} W}$ in dependence on the frequency for the LSM plasmon and for the TM plasmon is presented in Fig. 5b. One can see that the ratio ${P \mathord{\left/ {\vphantom {P W}} \right. \kern-\nulldelimiterspace} W}$ for the LSM plasmon grows near the cut-off frequency, due to greater energy release from graphene for the LSM plasmon compared to TM plasmon near LSM plasmon cut-off frequency. Note that the effect of this second factor on enhancing the power increment of the LSM plasmon is smaller compared to the decrease of the energy velocity of the LSM mode near its cut-off frequency.

**Figure 6:**
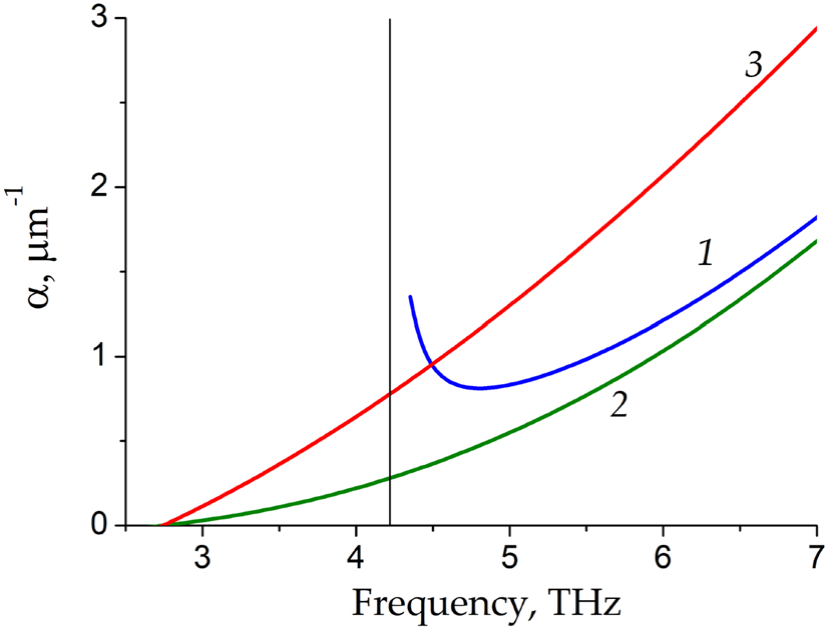
The power increment of the LSM plasmon for the groove width *L* = 1 μm (curve *1*) and power increments of the TM plasmons in layered graphene structures (curves *2*, *3*) in dependence on frequency. Curves *1* and* 2* correspond to the substrate thickness *d* = 10 μm, while curve *3* corresponds to *d* = 0.01 μm. Vertical straight thin line indicates the cut-off frequency of the LSM mode.

It is known that graphene screening by a nearby metal plate (gate) enhances the amplification of THz plasmons as compared to the case of unscreened plasmons due to stronger vertical confinement of the screened plasmon field near graphene^25^. Comparison the effect of vertical and lateral plasmon field confinement on the power increment of the LSM and TM plasmon modes is shown in Fig. 6. One can see from Fig. 6 that vertical screening of graphene enhances the THz plasmon amplification almost in the entire frequency range. However, the power increment of the LSM plasmon in the groove waveguide with the substrate thickness *d* = 10 μm (curve *1*) near its cut-off frequency is greater than the power increment of the TM plasmon in the layered graphene structure with a much closer metal screen placed at distance *d* = 0.01 μm from graphene (curve *3*). It means that the enhancement of the LSM plasmon power increment near its cut-off frequency is a stronger effect as compared to that due to screening of graphene.

Note that the energy velocity introduced in our paper well coincides with the group velocity in the systems with small losses (or small gain as considered in our paper)^26^. However, the effect of losses (gain) increases near the cut-off frequencies since the imaginary part of the wavevector become equal and can even exceed its real part (the latter tends to zero at the cut-off frequency). As a result, the group velocity loses definite physical meaning and senselessly differs from the energy velocity in this case. That is why we use only the energy velocity quantity in our paper.

## Methods

The spatio-temporal dependencies of the plasmon fields in the groove waveguide are assumed in the form $$\,e^{{ - i\omega t + ik_{x} x + ik_{y} y}} \cdot \left( {A^{ + } e^{{ik_{z} z}} + A^{ - } e^{{ik_{z} z}} } \right)$$ above graphene ($$A^{ + }$$ and $$A^{ - }$$ are the amplitudes of electric or magnetic fields of the forward and the counter-propagating waves, respectively, along *z* axis) and $$e^{{ - i\omega t + ik_{x} x}} \cdot \left[ {\left( {A^{ + + } e^{{ik_{z} z}} + A^{ + - } e^{{ik_{z} z}} } \right) \cdot e^{{ik_{y} y}} + \left( {A^{ - + } e^{{ik_{z} z}} + A^{ - - } e^{{ik_{z} z}} } \right) \cdot e^{{ - ik_{y} y}} } \right]$$ in the substrate ($$A^{ + + } ,\,$$$$A^{ + - } ,\,$$$$A^{ - + } ,\,$$ and $$A^{ - - }$$ are the amplitudes of fields of the forward and the counter-propagating waves along the *y* and *z* axes, where the first superscript indicates the direction along the *y* axis, the second index does for the *z* axis). We use the following boundary conditions. The components of the electric field tangential to the metal surfaces are set to zero at the walls and bottom of the groove waveguide: $$E_{x,z} \left( {y = - d} \right) = 0,$$ $$E_{x,y} \left( {z = 0} \right) = 0,$$ $$E_{x,y} \left( {z = L} \right) = 0.$$ The tangential components of the electric field are continuous across graphene: $$E_{{x{\text{a}}}} \left( {y = 0} \right) = E_{{x{\text{s}}}} \left( {y = 0} \right),E_{{z{\text{a}}}} \left( {y = 0} \right) = E_{{z{\text{s}}}} \left( {y = 0} \right).$$ The discontinuity of the tangential components of the magnetic field across graphene are equal to the current density components in graphene $$H_{{z{\text{a}}}} \left( {y = 0} \right) - H_{{z{\text{s}}}} \left( {y = 0} \right) = \sigma_{{{\text{gr}}}} \left( \omega \right) \cdot E_{x} \left( {y = 0} \right);$$$$H_{{x{\text{a}}}} \left( {y = 0} \right) - H_{{x{\text{s}}}} \left( {y = 0} \right) = - \sigma_{{{\text{gr}}}} \left( \omega \right) \cdot E_{z} \left( {y = 0} \right).$$ Finally, we derive the following dispersion equation for the LSM modes

2 $$\frac{{\varepsilon_{{\text{s}}} }}{{k_{{y\,{\text{s}}}} }}{\text{coth}}\;\left( {k_{{y\,{\text{s}}}} d} \right) + \frac{{\varepsilon_{{\text{a}}} }}{{k_{{y\,{\text{a}}}} }} = - \frac{{\sigma_{{{\text{gr}}}} \left( \omega \right)}}{{\omega \varepsilon_{0} }},$$

where $k_{{y\,{\text{a,s}}}} = \sqrt {\varepsilon_{{\text{a,s}}} \left( {{\omega \mathord{\left/ {\vphantom {\omega c}} \right. \kern-\nulldelimiterspace} c}} \right)^{2} - k_{x}^{2} - k_{z}^{2} }$ are the wavevector components normal to the graphene plane, the sign before the radical for the normal to graphene wavevector component above graphene is chosen to meet the condition of the surface wave, $k_{z} = {\pi \mathord{\left/ {\vphantom {\pi L}} \right. \kern-\nulldelimiterspace} L}$ is the transverse (along the *z* axis) wavevector component (we study the fundamental mode along *z* axis in this paper, only), ε_0_ is the electric constant, $\sigma_{{{\text{gr}}}} \left( \omega \right)$ is the dynamic conductivity of active graphene given by^6^:

3 $$\sigma_{{{\text{gr}}}} \left( \omega \right) = \frac{{e^{2} }}{4\hbar }\left\{ {\frac{{8ik_{{\text{B}}} T}}{{\pi \hbar {\kern 1pt} \left( {\omega + {i \mathord{\left/ {\vphantom {i \tau }} \right. \kern-\nulldelimiterspace} \tau }} \right)}}\ln } \right.\left( {1 + \exp \;\left( {\frac{{E_{F} }}{{k_{{\text{B}}} T}}} \right)} \right)\left. { + \tanh \;\left( {\frac{{\hbar {\kern 1pt} \omega - 2E_{F} }}{{4k_{{\text{B}}} T}}} \right) + \frac{4i\hbar \omega }{{\pi }}\int\limits_{0}^{\infty } {\frac{{G\left( {\varepsilon ,E_{F} } \right) - G\left( {{{\hbar {\kern 1pt} \omega } \mathord{\left/ {\vphantom {{\hbar {\kern 1pt} \omega } 2}} \right. \kern-\nulldelimiterspace} 2},E_{F} } \right)}}{{\left( {\hbar \omega } \right)^{2} - 4\varepsilon^{2} }}d\varepsilon } } \right\},$$

where *e* is the elementary charge, $\hbar$ is the reduced Planck constant, $k_{{\text{B}}}$ is the Boltzmann constant τ, and *T* are the mean free time and temperature of the charge carriers in graphene, respectively, $E_{{\text{F}}} > 0$ is the quasi-Fermi energy in graphene determining the inversion of the charge carriers (+ E_F_ and –E_F_ for electrons and holes, respectively), $G\left( {\varepsilon ,\varepsilon ^{\prime}} \right) = {{\sinh \;\left( {{\varepsilon \mathord{\left/ {\vphantom {\varepsilon {k_{{\text{B}}} T}}} \right. \kern-\nulldelimiterspace} {k_{{\text{B}}} T}}} \right)} \mathord{\left/ {\vphantom {{\sinh \;\left( {{\varepsilon \mathord{\left/ {\vphantom {\varepsilon {k_{{\text{B}}} T}}} \right. \kern-\nulldelimiterspace} {k_{{\text{B}}} T}}} \right)} {\left[ {\cosh \;\left( {{\varepsilon \mathord{\left/ {\vphantom {\varepsilon {k_{{\text{B}}} T}}} \right. \kern-\nulldelimiterspace} {k_{{\text{B}}} T}}} \right) + \cosh \;\left( {{{\varepsilon ^{\prime}} \mathord{\left/ {\vphantom {{\varepsilon ^{\prime}} {k_{{\text{B}}} T}}} \right. \kern-\nulldelimiterspace} {k_{{\text{B}}} T}}} \right)} \right]}}} \right. \kern-\nulldelimiterspace} {\left[ {\cosh \;\left( {{\varepsilon \mathord{\left/ {\vphantom {\varepsilon {k_{{\text{B}}} T}}} \right. \kern-\nulldelimiterspace} {k_{{\text{B}}} T}}} \right) + \cosh \;\left( {{{\varepsilon ^{\prime}} \mathord{\left/ {\vphantom {{\varepsilon ^{\prime}} {k_{{\text{B}}} T}}} \right. \kern-\nulldelimiterspace} {k_{{\text{B}}} T}}} \right)} \right]}}.$ The integral in the right-hand side of dynamic conductivity formula has to be interpreted as the principal value integral. The first term in the curly braces in Eq. (3) describes a Drude-model response for the intraband processes involving the phenomenological electron and hole scattering time *τ*. The remaining terms in Eq. (3) arise from the interband transitions. The second term in the curly braces becomes negative for $\hbar {\kern 1pt} \omega < 2E_{F}$ which corresponds to the population inversion in graphene. At sufficiently large $E_{F}$ (strong pumping), the interband stimulated emission of photons (plasmons) can prevail over the intraband (Drude) absorption. In this case, the real part of the dynamic conductivity of graphene can be negative in the THz range which leads the plasmon amplification.

Note that the dispersion expression for the TM mode in a layered graphene structure reads the same as Eq. (2) given there is only one wavevector component transverse to the plasmon propagation direction: $k_{{y\,{\text{a,s}}}} = \sqrt {\varepsilon_{{\text{a,s}}} \left( {{\omega \mathord{\left/ {\vphantom {\omega c}} \right. \kern-\nulldelimiterspace} c}} \right)^{2} - k_{x}^{2} .}$

**Figure 7:**
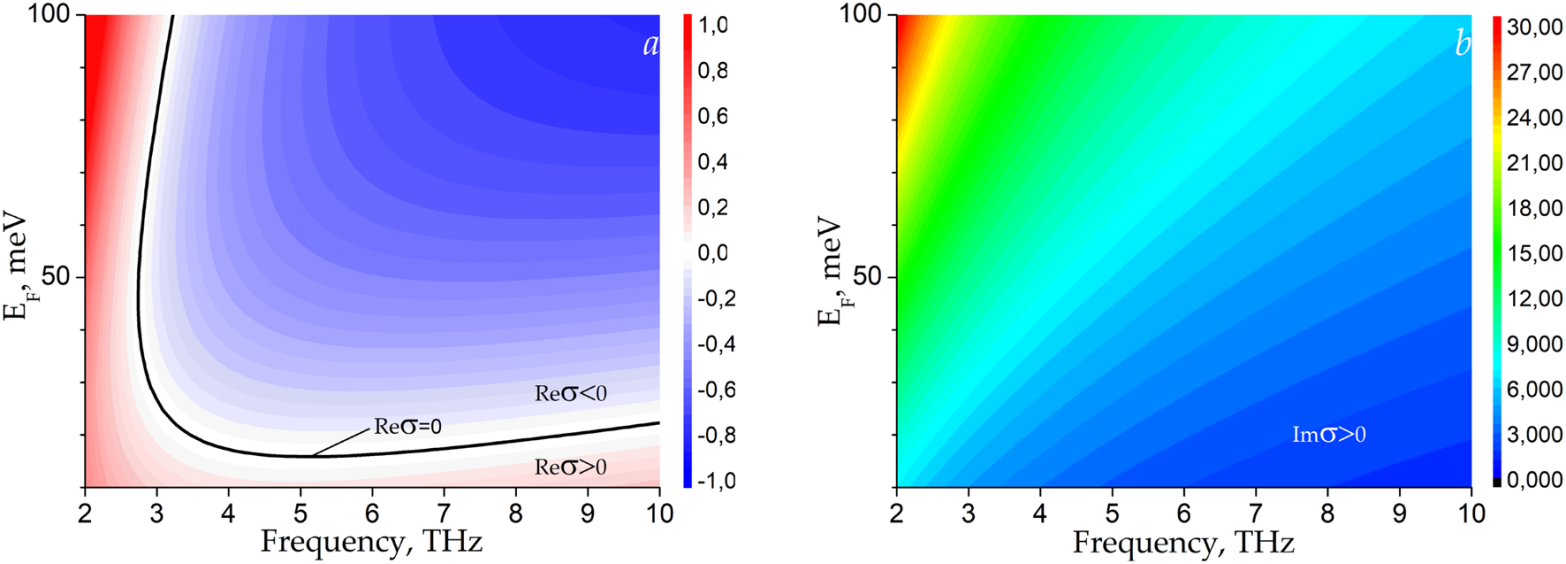
Color raster maps for the real (**a**) and imaginary (**b**) parts of the graphene conductivity calculated by Eq. (3) versus frequency and quasi-Fermi energy value.

Figures 7a and 7b show color raster maps with plots of Eq. (3) for the real and imaginary parts of graphene conductivity as the functions of frequency and Fermi energy for *τ* = 1 ps^27^. It is seen from Fig. 7a that the real part of the graphene conductivity can be negative at THz frequencies for $E_{{\text{F}}} > 20$ meV. The imaginary part of the graphene conductivity is positive (inductive) in the entire THz frequency range which is the necessary, as well as sufficient, condition for existing plasmons in graphene^28^.

Numerical solution of Eq. (2) was performed for the structure with the following.parameters: $\varepsilon_{{\text{s}}} = 5$ (corresponds to hBN), *T* = 300 K, *τ* = 1 ps, $E_{{\text{F}}} = 50$ meV, *d* = 10 μm unless different value is specified. These parameter values correspond to a negative real part of the graphene conductivity at THz frequencies yielding the possibility of THz plasmon amplification.

## Conclusions

In conclusion, the concept of THz waveguide plasmon amplifier based on a metal groove with active graphene is proposed. It is shown that the power amplification factor of the LSM waveguide plasmon (normalized to the plasmon wavelength) near the cut-off frequency of this mode can exceed that of the TM plasmon in a layered graphene structure by more than four orders of magnitude for the same frequency. This is caused by the increase of the LSM plasmon wavelength (up to tens of micrometer) and stronger interaction of the LSM plasmon with active graphene as compared to the TM plasmon due to smaller energy velocity of the LSM mode and greater energy release from graphene for the LSM plasmon near its cut-off frequency. The plasmon energy velocity exceeds 10^6^ m/s at the points of terminating the solid curves in Fig. 5a which is at least an order of magnitude greater than the electron transport velocity achievable in graphene. Therefore, the response operational speed of the plasmonic terahertz amplifier can be greater than that of the electronic counterpart (e.g., transistor). Terahertz waveguide plasmon amplifier can be used as an active element in terahertz plasmonic graphene nanocircuits.

## Data Availability

The data that support the findings of this study are available from the corresponding author upon request.
